# The Impact of the Closure of the Live Poultry Market due to COVID-19 on the Avian Influenza Virus in Nanchang, Jiangxi Province, China

**DOI:** 10.4269/ajtmh.21-0732

**Published:** 2021-10-29

**Authors:** Jin Guo, Wentao Song, Xiansheng Ni, Kun Zhou, Jingwen Wu, Wei Liu, Wen Xia, Fenglan He, Xiaoqin Tong, Guoyin Fan, Zhiqiang Deng, Zheng Liao, Haiying Chen, Shengen Chen

**Affiliations:** ^1^ The Collaboration Unit for Field Epidemiology of State Key Laboratory of Infectious Disease Prevention and Control, Jiangxi Provincial Key Laboratory of Animal-origin and Vector-borne Diseases, Nanchang Center for Disease Control and Prevention, Nanchang 330038, PR China;; ^2^ School of Public Health, Nanchang University, Jiangxi Provincial Key Laboratory of Preventive Medicine, Nanchang University, Nanchang 330006, PR China

## Abstract

This article aims to understand the changes in the detection rates of H5, H7, and H9 subtypes of avian influenza viruses (AIVs) in the live poultry markets (LPMs) in Nanchang City, Jiangxi Province, before and after the outbreak of the COVID-19. From 2019 to 2020, we monitored the LPM and collected specimens, using real-time reverse transcription polymerase chain reaction technology to detect the nucleic acid of type A AIV in the samples. The H5, H7, and H9 subtypes of influenza viruses were further classified for positive results. We analyzed 1,959 samples before and after the outbreak and found that the positive rates of avian influenza A virus (39.69%) and H9 subtype (30.66%) after the outbreak were significantly higher than before the outbreak (26.84% and 20.90%, respectively; *P* < 0.001). In various LPMs, the positive rate of H9 subtypes has increased significantly (*P* ≤ 0.001). Positive rates of the H9 subtype in duck, fecal, daub, and sewage samples, but not chicken samples, have increased to varying degrees. This study shows that additional measures are needed to strengthen the control of AIVs now that LPMs have reopened after the relaxing of COVID-19–related restrictions.

## INTRODUCTION

Influenza A virus is a single-stranded negative-sense RNA virus that consists of eight gene fragments.^1^ So far, 18 hemagglutinins (HA; H1–H18) and 11 neuraminidase (NA; N1–N11) have been found.^2^ Since 1959, highly pathogenic H5 and H7 subtype avian influenza viruses (AIVs) carrying different NA subtypes have caused a large number of disease outbreaks in poultry and wild birds worldwide.^3^ In addition, new recombine and virus variants continue to emerge, including H7N3, H9N9, H9N6, and H5N8 subtypes.^4^ Highly pathogenic avian influenza viruses (HPAIV) H5N6 and H7N9 are still mutating. They continue to erupt in poultry and wild birds in parts of China, and they have also caused continuous poultry death in other countries.^4^^,^^5^ As of May 26, 2021, in China, there were 81 human infections of H5 (H5N1 and H5N6), 1,537 human infections of H7 (H7N9), and 62 human infections of H9 (H9N2).^6^ Therefore, we must still stringently monitor the H5, H7, and H9 subtypes.

Since December 2019, there have been outbreaks of COVID-19 around the world. China quickly took preventive and control measures against the COVID-19 outbreak, and many places have completely or partially closed live poultry trading markets. Nanchang also closed the live poultry markets (LPMs) at the end of January 2020. Nanchang has a unique geographic location; it is located not only in southern China, which is regarded as the theoretical center of the potential influenza virus outbreak,^7^ but also is near Poyang Lake, China’s largest freshwater lake. Poyang Lake is an important wintering place for migratory birds from East Asia to Australia,^8^ and the birds have frequent contact with poultry raised by farmers nearby.^9^ Led by the Nanchang Bureau of Agriculture and Rural Affairs, the city opened LPMs in late March after the COVID-19 outbreak was brought under control. This study sampled and analyzed the changes of AIVs in the LPM before and after the COVID-19 outbreak in Nanchang City, Jiangxi Province, China, to understand the changes in AIVs before and after the outbreak and provide a basis for further prevention and control measures.

## MATERIALS AND METHODS

### Ethics approval and consent to participate.

This study was approved by the Institutional Review Board of Nanchang Center for Disease Control and Prevention. The institute did not issue a number or ID card for the animal study because the poultry studied was not an endangered or protected species. Biological samples were collected directly from the oropharynx and cloaca of healthy chickens and ducks with the oral consent of poultry breeders.

### Fieldwork.

Generally, the period from January to March 2020 is considered the outbreak period of China’s COVID-19 epidemic. During this period, many provinces and cities in China experienced the control and deregulation of roads and communities. At the beginning of the outbreak in January 2020, many prefectures and cities began to close LPMs. Nanchang also closed the LPM at this time then opened various LPMs one at a time at the end of March. In this study, before the outbreak of the COVID-19 in 2019 and after the outbreak in 2020, we use a stratified random sampling method to select four counties and districts including Donghu, Xihu, and Qingshanhu districts and Xinjian county. We then choose an LPM in each county district as the site for annual monitoring, and all four selected markets were sampled every 2 months from February 2019 to December 2020. Because of the impact of the epidemic, we did not conduct sampling in February 2020. There are fewer live poultry stalls in retail markets than wholesale markets, and therefore we chose three retail markets and one wholesale market. In the live poultry retail market, the poultry sold at each stall is more complicated and includes many kinds of live poultry, such as chickens, ducks, and geese; live poultry slaughtered on-site are also sold, and there are more customers. However, in the wholesale market, only live poultry is sold, not slaughtered poultry; the live poultry sold in each stall is generally of one type; and there are fewer customers than in the retail market. For each sampling, multiple poultry sales stalls were selected in each LPM, seeking to include, if possible, all the stalls selling poultry in the market. We used sterile cotton swabs provided with commercial virus tubes to collect environmental and live poultry samples.

### Viral analysis.

The samples were immediately sent to the laboratory of Nanchang Center for Disease Control and Prevention at 4°C after collection and retained in the storage medium. According to the manufacturer’s instructions. Viral RNA was extracted from biological samples with QIAamp Viral RNA Mini Kit (Qiagen, Hilden, Germany). Real-time reverse transcriptase polymerase chain reaction (RT-PCR) was used for influenza typing and subtyping. The samples were identified as containing influenza A on the basis of the M gene but could not be classified into subtypes. Specific real-time RT-PCR assays for avian influenza H5, H7, and H9 were done to verify the viral subtypes from nucleic acids positive to influenza A virus using the Influenza Virus A Real Time RT-PCR Kit (Lifeliver, Shanghai, China).

### Statistical analysis.

The count data are expressed in frequency and percentage. Because we needed to detect the significance of the difference between the positive rates of two or more poultry samples, we used SPSS 22.0 to conduct χ^2^ tests on the input data after data entry into Excel to understand the difference between positive rates. When *P* < 0.05, the difference was statistically significant.

## RESULTS

### Detection of AIVs before and after COVID-19 outbreak.

**Figure 1.**
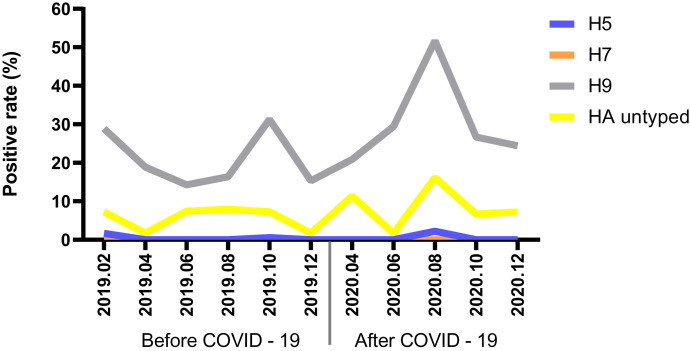
Changes of avian influenza virus subtypes before and after the COVID-19 outbreak. This figure appears in color at www.ajtmh.org.

We collected 1,062 and 897 samples before and after the COVID-19 outbreak. The positive rate of avian influenza A virus detected after the outbreak (39.69%) was significantly higher than that before the outbreak (26.84%; *P* < 0.001). Before and after the COVID-19 outbreak, the H5 and H9 subtype infections were all detected except for the H7 subtype. It is worth noting that after the outbreak, the positive rate of H9 subtype (30.66%) and the positive rate of untypable HA type (8.58%) increased significantly (*P* < 0.01) (Table 1). Before COVID-19, the positive rate of H5 and H7 subtypes remained low with little change. In October 2019, the positive rate of H9 subtype (31.28%) reached a peak. Comparing the same months before and after the outbreak, the positive rate of avian influenza A virus only decreased slightly in October (*P* > 0.05) and then increased the rest of the month (*P* < 0.05). In August 2020, the positive rates of H5 (2.22%), H9 (51.67%), and untypable HA subtypes (16.11%) reached their peaks during the entire observation period (Figure 1).

**Table 1:** The positive rate (%) of avian influenza virus before and after the COVID-19 outbreak

Period	*N*	No. positive (%)	HA Subtype (%)			
			H5	H7	H9	HA untyped
Before the epidemic	1,062	285 (26.84)	4 (0.38)	0 (0)	222 (20.9)	59 (5.56)
After the epidemic	897	356 (39.69)	4 (0.45)	0 (0)	275 (30.66)	77 (8.58)
Total	1,959	641 (32.72)	8 (0.41)	0 (0)	497 (25.37)	136 (6.94)
χ^2^		36.484	0.057		24.434	6.904
*P*		0.000	1.000		0.000	0.009

### Detection results in different types of samples.

**Figure 2.**
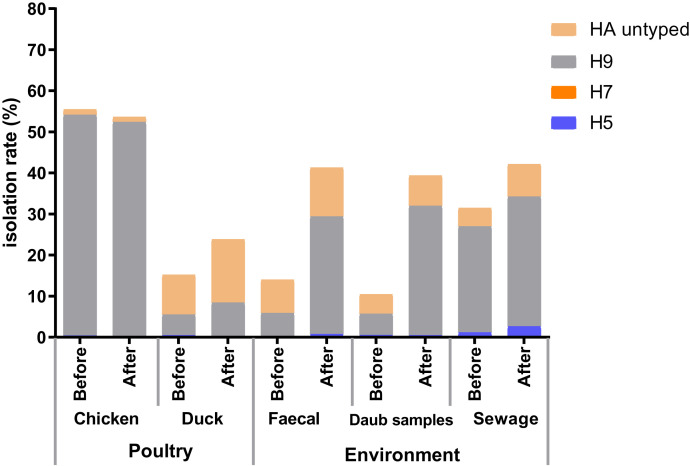
The difference in the positive rate (%) of avian influenza virus among samples before and after the COVID-19 outbreak. This figure appears in color at www.ajtmh.org.

Before and after the COVID-19 outbreak, we collected 540 and 462 poultry samples (throat swabs and cloacal samples of chickens and ducks), among which the positive rates of avian influenza A virus were 37.78% and 38.96%, respectively. We collected 522 and 435 environmental samples (poultry fecal, surface wipes of cages, and sewage from poultry cleaning) before and after the outbreak, among which the positive rate of avian influenza A virus detected after the outbreak (40.46%) was significantly higher than that before the outbreak (15.52%; *P* < 0.001), and the positive rate of H9 (30.57%) also increased significantly (*P* < 0.001). After the outbreak, there was no significant difference in the changes in the positive rates of different subtypes in poultry samples (Table 2). Further analysis found that, except for chicken samples, the positive rate of the H9 subtype in the remaining samples (duck, fecal, daub, and sewage samples) increased to varying degrees. Among environmental samples, the positive rates of the H9 subtype in daub samples (31.48%) and fecal samples (28.67%) after the outbreak were significantly higher than before the outbreak (5.21% and 5.86%, respectively; *P* < 0.001) (Figure 2).

**Table 2:** Avian influenza virus test results of poultry and environmental samples before and after the outbreak

Period	*N*	No. positive (%)	HA subtype (%)			
			H5	H7	H9	HA untyped
Poultry	1,002	384 (38.32)	2 (0.20)	0 (0)	317 (31.64)	65 (6.49)
Before COVID-19	540	204 (37.78)	2 (0.37)	0 (0)	175 (32.41)	27 (5.00)
After COVID-19	462	180 (38.96)	0 (0)	0 (0)	142 (30.74)	38 (8.23)
Environmental	957	257 (26.85)	6 (0.63)	0 (0)	180 (18.81)	71 (7.42)
Before COVID-19	522	81 (15.52)	2 (0.38)	0 (0)	47 (9.00)	32 (6.13)
After COVID-19	435	176 (40.46)	4 (0.92)	0 (0)	133 (30.57)	39 (8.97)
Total	1,959	641 (32.72)	8 (0.41)	0 (0)	497 (25.37)	136 (6.94)

HA = hemagglutinins.

### Detect results of different LPMs.

Before and after the COVID-19 outbreak, we collected 540 and 450 samples from the live poultry wholesale market and 522 and 447 samples from the live poultry retail market. Before the epidemic, the positive rate in the wholesale market (25.19%) was similar to that in the retail market (28.54%), but after the epidemic, the positive rate in the wholesale market (45.33%) was significantly higher than that in the retail market (34.00%; *P* = 0.001). In the wholesale market, the positive rate of avian influenza A virus (45.33%) detected after the outbreak increased (*P* < 0.001), and the positive rate of H9 subtype (28.89%) also increased (*P* = 0.001). In the retail market, the positive rate of avian influenza A virus (34.00%) increased slightly after the outbreak, and the positive rate of the H9 subtype (32.44%) increased significantly (*P* < 0.001; Table 3).

**Table 3:** Avian influenza virus test results in wholesale and retail markets before and after the outbreak

Period	*N*	No. positive (%)	HA subtype (%)			
			H5	H7	H9	HA untyped
Wholesale market	990	340 (34.34)	0 (0)	0 (0)	236 (23.84)	104 (10.51)
Before COVID-19	540	136 (25.19)	0 (0)	0 (0)	106 (19.63)	30 (5.56)
After COVID-19	450	204 (45.33)	0 (0)	0 (0)	130 (28.89)	74 (16.44)
Retail market	969	301 (31.06)	8 (0.83)	0 (0)	261 (26.93)	32 (3.3)
Before COVID-19	522	149 (28.54)	4 (0.77)	0 (0)	116 (22.22)	29 (5.56)
After COVID-19	447	152 (34.00)	4 (0.89)	0 (0)	145 (32.44)	3 (0.67)
Total	1,959	641 (32.72)	8 (0.41)	0 (0)	497 (25.37)	136 (6.94)

HA = hemagglutinins.

## DISCUSSION

Through active monitoring of LPMs in Nanchang before and after the COVID-19 outbreak, we found that after the outbreak, the positive rate of avian influenza A virus showed an upward trend. On the one hand, probably due to the sudden outbreak of the COVID-19 epidemic, LPMs in many cities across the country were forced to close urgently, roads were also blocked, logistics was hindered, socially concentrated activities were cancelled, consumer demand declined, and live poultry in farms could not be sold. As a result, live poultry culling was affected, and a large number of live birds were housed together, resulting in the rapid spread of the virus among live birds. On the other hand, the sudden closure of the LPMs affected by the outbreak may have resulted in a backlog of poultry in other temporary holding facilities, where poultry from different regions are mixed; this will also increase the presence of the virus before reentering the market. Studies have also pointed out that during the closure of the LPMs, the detection rate of avian influenza dropped significantly, but once the LPMs reopened, the detection rate would be as high or even higher than before the closure.^10^ Because of the impact of the COVID-19 epidemic, the LPMs were forced to close for as long as 2 months. In the past, it was generally closed for shorter periods of about 2 weeks. The results of our and previous studies^11^ show that no matter whether it is a short-term or long-term market closure, the effect on controlling the spread of AIV among live birds is not clear. However, from the perspective of human-to-human transmission of the AIV, in areas where the virus is found in live poultry or people, the rapid implementation of LPM closures has a relatively good control effect in the short term.^12^^,^^13^

After the LPM reopened, to lessen the risk of transmission of the virus to humans, the retail market reduced the sale of live poultry, instead selling mainly slaughtered poultry. Therefore, the positive rate of AIV in the retail market after the epidemic is lower than that in the wholesale market. This also confirms that the establishment of a live poultry slaughter center proposed by research^14^ is effective in reducing the spread of AIV among live poultry. We have also noticed that the detection rate of AIV in environmental samples has increased significantly after the outbreak. On the one hand, it may be because the LPM lifted the 2-month lockdown. In the following period, the public’s demand for live poultry increased, as did the circulation of live poultry, leading to an increase in AIV in the environment. On the other hand, this increase may be due to insufficient environmental cleaning and disinfection after the LPM reopened. One study showed^15^ that regular market closures, disinfection, and cleaning are influencing factors related to AIV contamination, and other studies have shown^16^^,^^17^ that regular market closures, disinfection, and cleaning can minimize pollutants in the LPMs. In our research, we reported for the first time the changes in AIV in the LPM before and after the COVID-19 pandemic, which provides a reference for similar studies. Further investigation is required to determine the underlying reasons for the increased environmental contamination after the LPM was reopened after the outbreak. Therefore, in the future, we hope to have more evidence to explain the changing trend in environmental AIV after the reopening of the LPMs after the epidemic. In different environmental samples, the rate of AIV-positive daub samples was significantly higher than before the outbreak. The daub samples are collected from surfaces such as processing tables, cutting boards, and chicken coops, which poultry workers or customers often touch. This increase suggests that poultry workers and customers are often exposed to AIV after the outbreak. It has been reported^18^ that some infected patients, without direct or indirect contact with poultry, simply went to an LPM but were infected with AIV. Further, in the poultry market, people have more opportunities to be exposed to the environment. Therefore, compared with poultry infected with AIV, an environment with a high viral load is more likely to pose a threat to poultry workers and customers. Our results show that daub samples are more sensitive than other environmental samples, which is consistent with other studies.^10^ Therefore, when disinfecting an LPM, the main source of virus contamination in the environment should be considered first to improve the efficiency of disinfection.

H9 is the main subtype detected in all samples and markets before and after the outbreak. Studies have pointed out that the H9 (H9N2) subtype has replaced H5 (H5N6) and H7 (H7N9) as the main AIV subtype in Chinese chickens and ducks.^4^ We noted that in the two types of LPMs and their environmental samples, the detection rate of the H9 subtype has increased significantly after the outbreak, and mixed infections in markets are common. As new viral reassortants and variants of the H9 subtype continue to appear, including the H9N9 and H9N6 subtypes.^4^ Therefore, our high detection rate of H9 in all markets after the outbreak has undoubtedly increased the risk of H9 subtype genetic recombination to produce new influenza viruses. In addition, after 2009, the seroprevalence of H9 (H9N2) AIV among poultry workers in China has shown an upward trend.^19^ In fact, sporadic human cases caused by H9N2 are still being reported, and human cases caused by H9N2 have far exceeded those caused by H5N6 and H7N9 in recent years. In China, in just the first half of 2021, there were nine human cases of H9N2 infection, whereas only one case of H5N6 occurred, and there were no H7N9 infections.^6^ Importantly, a 2020 study showed that almost all the H9 AIVs encode HAs that preferentially bind to the human α2,6-linked sialic acid cell receptor.^20^ This indicates an increased tendency for human infection. Because of the innate recombination ability of AIV, the enhancement of human receptor binding ability, and the increasingly higher detection rate of H9 subtype, surveillance of AIV in poultry farms, LPMs, and wild birds should be restored to pre-COVID-19 levels or higher.

## CONCLUSIONS

Our data show that after the outbreak, the positive rate of avian influenza A virus and the H9 subtype increased significantly, and the performance was more obvious in environmental samples. This suggests that the environment in the LPM is not sufficiently clean after the COVID-19 outbreak. In response to this situation, decision-makers should consider strengthening the management of China’s LPMs after the epidemic, and it is important to adopt more comprehensive and effective prevention and control measures to reduce the AIV content in LPMs.
